# Drug-Induced Leukocytoclastic Vasculitis From an Unreported Source: Daptomycin

**DOI:** 10.7759/cureus.82490

**Published:** 2025-04-18

**Authors:** Jennifer K Priessnitz, Timothy Kuzel, Lindsay Ackerman, Nelson Nicolasora

**Affiliations:** 1 Internal Medicine, Joan and Macon Brock Virginia Health Sciences, Old Dominion University, Norfolk, USA; 2 Internal Medicine, University of Arizona College of Medicine - Phoenix, Phoenix, USA; 3 Dermatology, Medical Dermatology Specialists, Phoenix, USA; 4 Infectious Disease, University of Arizona College of Medicine - Phoenix, Phoenix, USA

**Keywords:** antibiotic adverse effects, cutaneous drug reaction, daptomycin adverse reaction, drug-induced vasculitis, histopathology of vasculitis, immune complex-mediated vasculitis, leukocytoclastic vasculitis (lcv), mrsa endocarditis, small vessel vasculitis

## Abstract

Leukocytoclastic vasculitis (LCV) is a rare small-vessel vasculitis caused by immune-complex-mediated deposition on endothelial cells of dermal capillaries. Common triggers include medications, infections, autoimmune disorders, and malignancies. We present a case of a 58-year-old male who developed daptomycin-induced LCV after being treated for Methicillin*-*resistant* Staphylococcus aureus* (MRSA) endocarditis. The patient experienced painful palpable purpura on his lower extremities, which was diagnosed as LCV by skin biopsy. At the time of the biopsy, warfarin and daptomycin were discontinued, and the patient was transitioned to heparin and ceftaroline. MRSA-induced LCV was ruled out, due to negative blood cultures and adequate source control of his infection at the time of development of the skin lesions. Warfarin-induced skin necrosis (WISN) was ruled out based on histopathological findings. This case is clinically significant as it represents the first reported case of LCV associated with daptomycin use. It underscores the importance of considering the patient's history, clinical presentation, and histopathological findings to ensure prompt recognition and management of this rare drug reaction, allowing for the resolution of LCV.

## Introduction

Leukocytoclastic vasculitis (LCV) is a small-vessel vasculitis arising from immune complex deposition on dermal capillary endothelium [1]. This results from various etiologies, including drug reactions, infections, rheumatologic disorders, connective tissue diseases, autoimmune conditions, malignancies, or idiopathically [2]. LCV is a rare condition, with an annual incidence of approximately 45 per million individuals. It affects both genders equally and can present at any age, although it typically presents in adults [2]. The clinical presentation of LCV can overlap with several conditions, making the diagnosis challenging. Common mimickers include drug eruptions, infectious emboli from endocarditis, cellulitis, viral exanthems, warfarin-induced skin necrosis (WISN), and antiphospholipid syndrome [3]. Therefore, obtaining a thorough history, performing a detailed physical examination, and conducting an appropriate diagnostic workup are critical for a prompt diagnosis and treatment.

We present the case of a 58-year-old male with acute Methicillin*-*resistant *Staphylococcus aureus* (MRSA) endocarditis secondary to osteomyelitis and bacteremia, who developed painful bilateral lower extremity purpura consistent with daptomycin-induced LCV, after 21 days of treatment.

This article was previously presented as a poster presentation at the 2025 AAAAI/WAO Joint Congress Annual Meeting on March 1, 2025.

## Case presentation

A 58-year-old male, with a past medical history of type II diabetes mellitus, underwent a right lower extremity fifth-digit osteotomy secondary to osteomyelitis. He was discharged with a 10-day course of empiric cephalexin. However, two weeks later, he was readmitted to the hospital with sepsis. Blood cultures were positive for MRSA, and imaging revealed osteomyelitis of the left calcaneus. The patient was initiated on piperacillin-tazobactam, vancomycin, and intravenous (IV) fluids. Following the incision and drainage by the podiatry team, a bone biopsy was negative for acute osteomyelitis. The patient's hospital course was complicated by the development of a 1.3 cm anterior leaflet mitral valve vegetation with multiple mobile projections consistent with MRSA endocarditis, as seen on transesophageal echocardiogram (TEE). Despite vancomycin, the patient had persistent bacteremia, prompting the change of IV antibiotics to IV daptomycin and ceftaroline. Following five days of combination therapy, repeat blood cultures had no growth, ceftaroline was discontinued, and daptomycin monotherapy was continued. The patient underwent mechanical mitral valve placement performed by cardiothoracic surgery. There was a consequent bridging from heparin to warfarin on postoperative day three.

**Figure 1:**
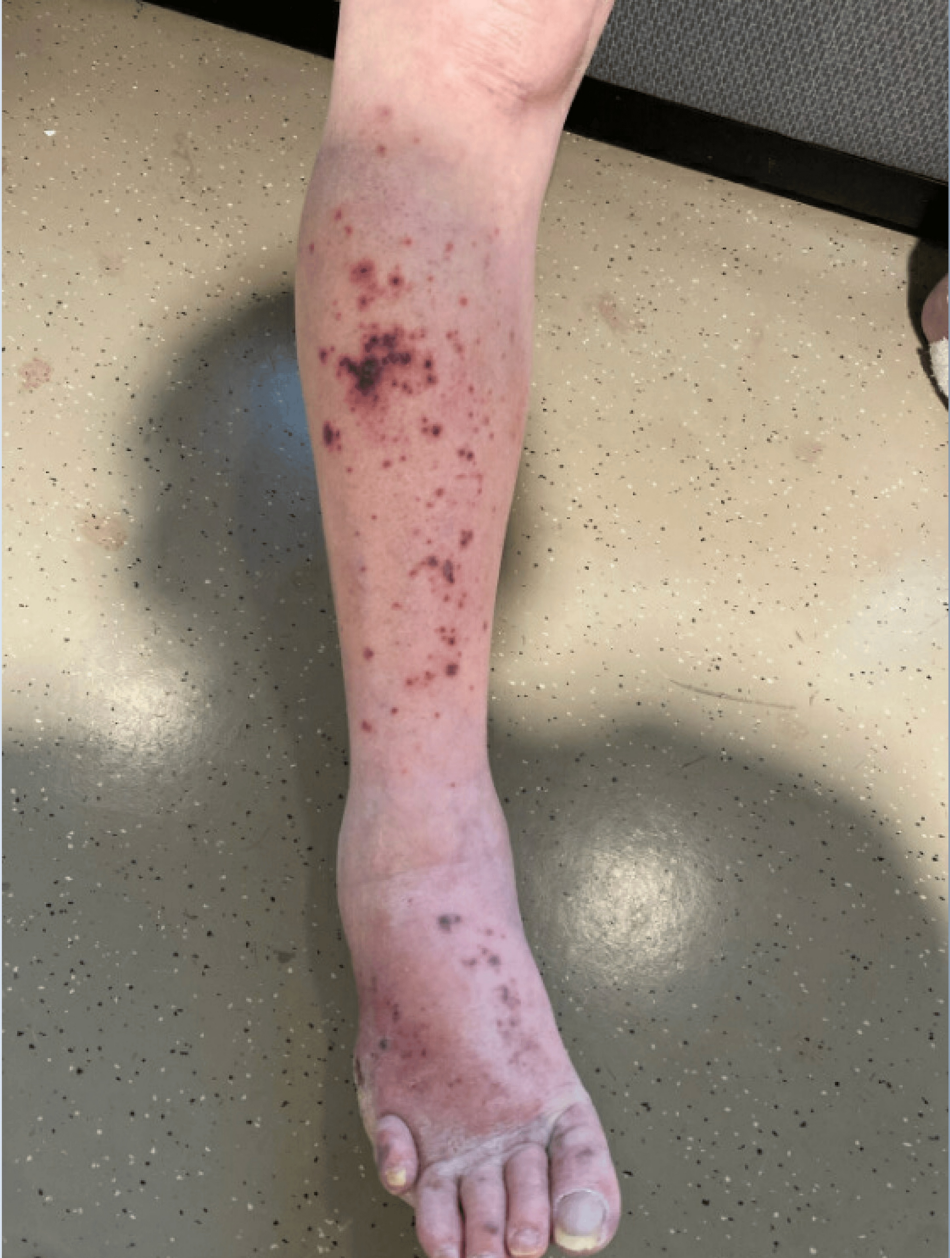
Right lower extremity painful, necrotic appearing purpura with surrounding erythema.

**Figure 2:**
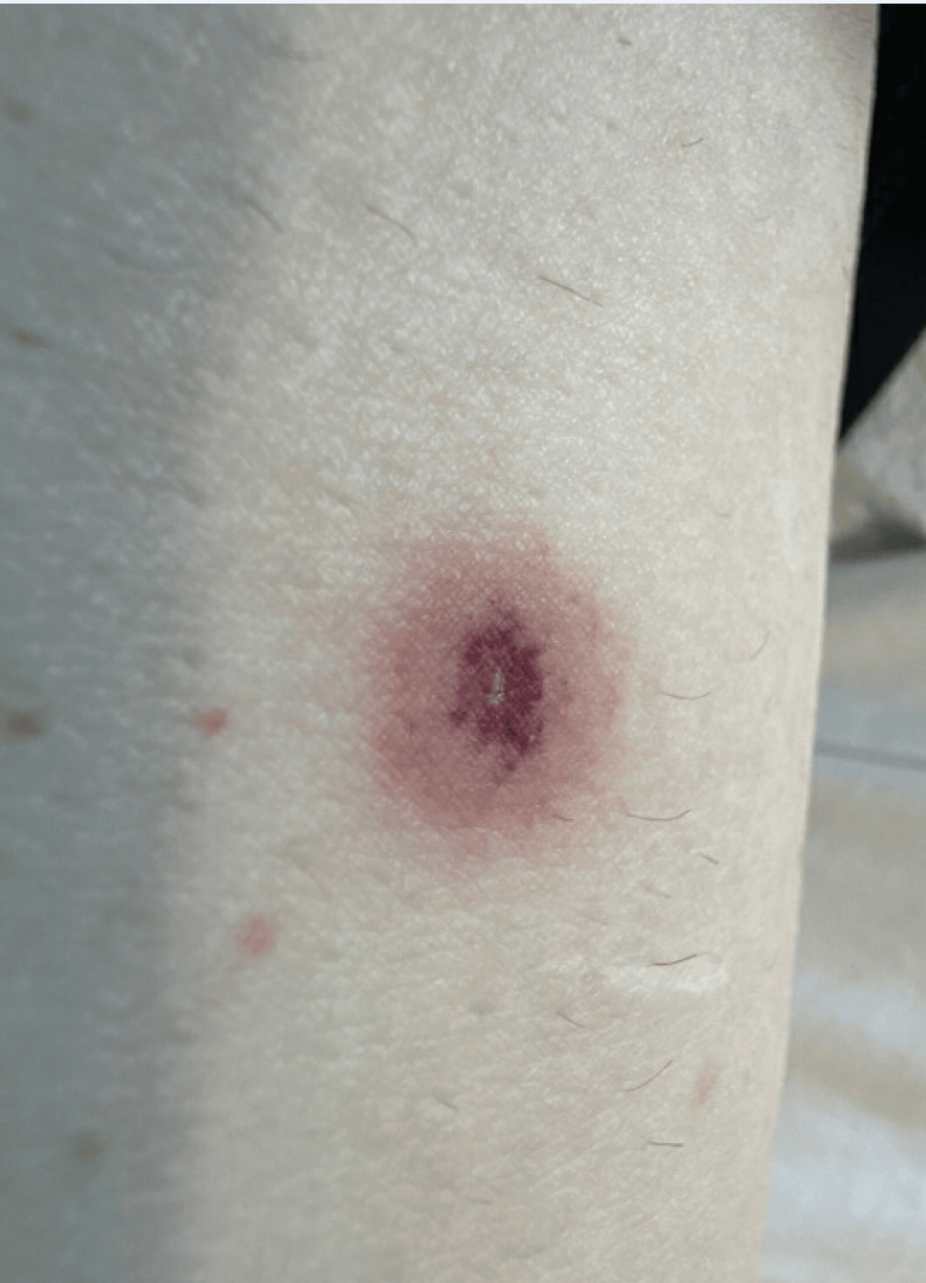
Erythematous macule with palpable purpura on the right lower extremity.

On postoperative day 10 and 21 days of daptomycin therapy, the patient developed right lower extremity pain (rated 8/10), necrotic-appearing 1-2 cm purpura with surrounding erythema on his right lower extremity. This subsequently extended to involve his right thigh and left leg (Figure 1). Warfarin was discontinued due to concern for WISN, and heparin was restarted until further evaluation could be performed. Vitamin K was administered to reverse the effects of warfarin. Daptomycin was also discontinued due to concerns for medication-induced LCV, and the patient was initiated on ceftaroline. Dermatology was consulted to evaluate for WISN versus medication-induced LCV. The dermatology team favored a diagnosis of LCV, which was confirmed with skin biopsies. MRSA-induced LCV was ruled out, due to negative blood cultures and adequate source control of the patient's infection before the development of his characteristic skin lesions (Figure 2). Daptomycin-induced LCV was singled out as the most likely culprit of LCV due to the timing of the skin eruptions relative to the initiation of daptomycin and the lack of other potential causes after reviewing the patient's clinical course and medications. Specifically, the lesions present were those of palpable purpura, distributed predominantly on lower extremities with an ascending distribution, within the typical 7-21-day window after initiation of a new medication [2]. The presentation was inconsistent with WISN, which typically occurs after three to five days of warfarin initiation and presents as non-palpable purpura without erythema on areas of the body with ample subcutaneous structures [4].

**Figure 3:**
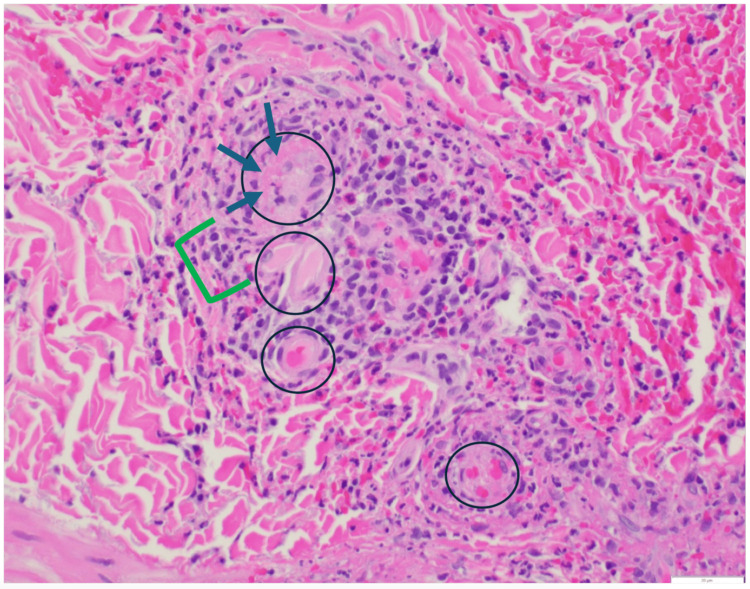
Biopsy of the right lower extremity demonstrating LCV. The black circles demonstrate vessels with inflammation marked by fibrin deposition and neutrophil invasion. The green bracket highlights the area of neutrophils (polymorphonuclear cells) and fragmentations of neutrophils (leukocytoclasia). Blue arrows mark eosinophilic regions representing fibrin deposition on vessels.

A punch biopsy of the purpuric lesions confirmed the LCV diagnosis. Histological examination revealed a dense, mixed perivascular infiltrate composed of lymphocytes, neutrophils, leukocytoclastic debris, and scattered eosinophils within the superficial to deep dermis. There was fibrinoid necrosis of vessel walls with prominent red blood cell extravasation, consistent with LCV (Figure 3). Direct immunofluorescence was negative, excluding Henoch-Schönlein purpura. Additionally, tissue cultures were negative, ruling out the possibility of infectious or MRSA-induced LCV. A comprehensive laboratory workup, including a hepatitis panel, cryoglobulin screen, antinuclear antibody (ANA), double-stranded DNA antibody (dsDNA), and antineutrophil cytoplasmic antibody (ANCA) panel, was unremarkable (Table 1). This ruled out an underlying infectious, autoimmune, or malignant etiology, supporting the diagnosis of daptomycin-induced LCV.

**Table 1:** Patient laboratory workup for vasculitis evaluation. HIV: human immunodeficiency virus; ANA: antinuclear antibody; dsDNA: double-stranded DNA; PR3-ANCA: proteinase 3-antineutrophil cytoplasmic antibody; MPO-ANCA: myeloperoxidase-antineutrophil cytoplasmic antibody; LCV: leukocytoclasitc vasculitis

Test	Result	Reference Range	Clinical Interpretation
C3 Complement	139 mg/dL	90-180 mg/dL	Normal
C4 Complement	47 mg/dL	10-50 mg/dL	Normal
Hepatitis A IgM	Negative	Negative	No active infection
Hepatitis B Core Antibody	Negative	Negative	No active infection
Hepatitis C Antibody	Negative	Negative	No active infection
Cryoglobulin	Negative	Negative	Negative
HIV-1/HIV-2 Antibody	Negative	Negative	No active infection
ANA	Negative	Negative	No autoimmune disease suggested
DsDNA	Negative	Negative	No active lupus
PR3-ANCA	Negative	Negative	No evidence of granulomatosis with polyangiitis
MPO-ANCA	Negative	Negative	No evidence of microscopic polyangiitis
Heparin-Induced Thrombocytopenia antibody	Negative	Negative	No evidence of Heparin-Induced Thrombocytopenia
Direct Immunofluorescence	Negative	Negative	No evidence of Henoch-Schönlein purpura
Skin Biopsy	Consistent with LCV	N/A	Confirmed diagnosis

**Figure 4:**
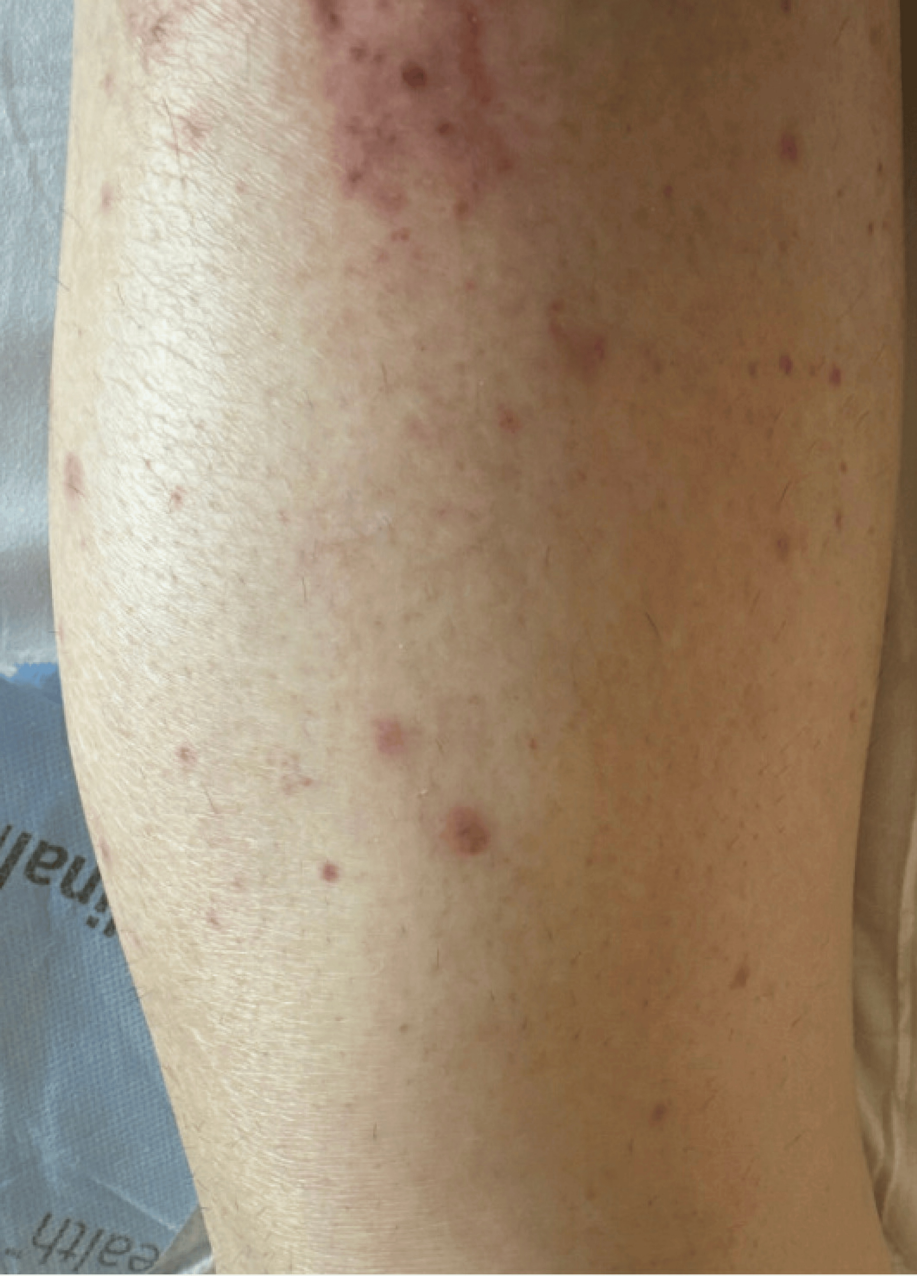
Patient’s right lower extremity at the one-month follow-up visit after discontinuation of daptomycin revealing resolution of purpuric lesions, with post-inflammatory changes of dyspigmentation and crusted re-epithelializing tissue being present.

**Figure 5:**
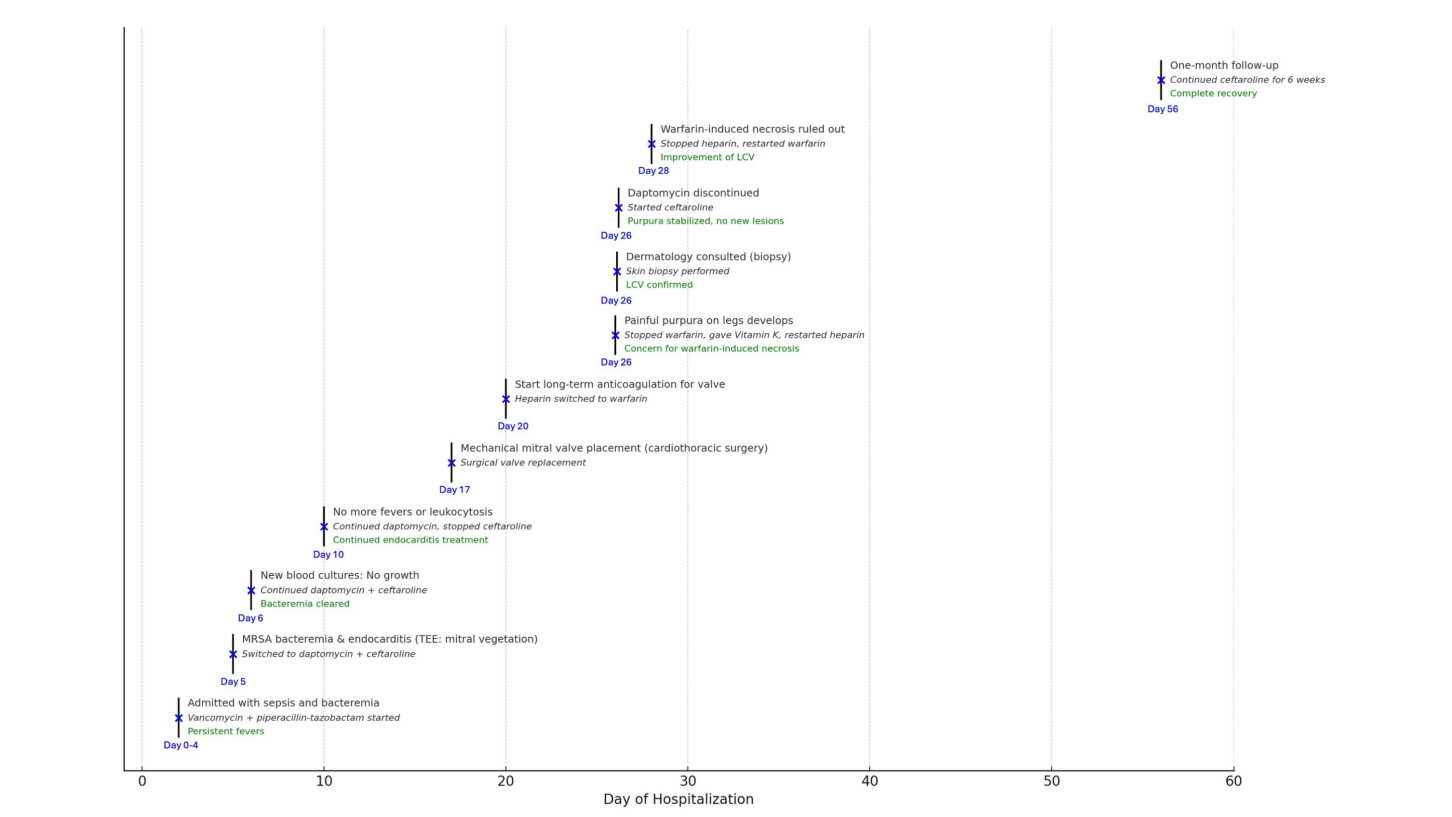
Timeline of the patient's clinical events.

Following confirmation of LCV, daptomycin was discontinued, and the patient was restarted on ceftaroline for continued treatment of MRSA endocarditis. The patient showed limited progression of purpura after the removal of daptomycin. After biopsy confirmation, warfarin was resumed without further evidence of skin necrosis. At the one-month follow-up visit, the patient's LCV lesions had resolved, with only post-inflammatory changes of dyspigmentation and crusted re-epithelializing tissue being present (Figure 4). A timeline of the patient's clinical course is seen in Figure 5.

## Discussion

LCV consists of palpable purpura, most commonly observed in dependent areas, such as the lower extremities, feet, and hands. It generally develops within seven to 21 days of drug initiation [2]. The pathogenesis of LCV involves cell-mediated and humoral immune responses, resulting in small vessel inflammation characterized by neutrophilic infiltration and degradation (leukocytoclasia) within the first 48 hours, followed by a transition to lymphocytic infiltration. This leads to fibrinoid necrosis of the vessel walls, with or without IgA deposition [2]. The etiology of LCV is broad, and all potential triggers should be carefully evaluated. Post-infectious LCV is commonly associated with pathogens such as *Streptococcus* spp., *Mycobacterium* spp., *Staphylococcus aureus, Chlamydia* spp., *Neisseria* spp., human immunodeficiency virus (HIV), hepatitis B, hepatitis C, and *Treponema pallidum* [2]. Medication-induced LCV is frequently triggered by antibiotics (e.g., beta-lactams, vancomycin, sulfonamides), antiepileptics (e.g., phenytoin), and other drugs such as allopurinol, NSAIDs, and furosemide, among many others [2,5-16]. Additionally, neoplastic LCV has been associated with malignancies such as lymphomas, leukemias, intestinal adenocarcinoma, and lung cancer. Systemic disease-associated LCV may occur in the context of connective tissue diseases (e.g., systemic lupus erythematosus, Sjögren's syndrome), inflammatory bowel disease, Behçet's disease, rheumatoid arthritis, cryoglobulinemic vasculitis, Henoch-Schönlein purpura, hypocomplementemic urticarial vasculitis, and erythema elevatum diutinum [2].

A comprehensive review of the current literature (conducted via PubMed, Embase, and Google Scholar) revealed that, to date, there are no documented cases of daptomycin-induced LCV. The most prominent adverse effects of daptomycin reported in the literature include rare occurrences of eosinophilic pneumonia and some cases of rhabdomyolysis, requiring creatine phosphokinase serology monitoring. However, according to the U.S. Food and Drug Administration drug labeling, LCV is not listed as a documented adverse reaction to daptomycin [17]. In our case, the temporal association between daptomycin initiation and the onset of palpable purpura, histopathological confirmation of LCV with eosinophils present, and negative tissue cultures strongly support a diagnosis of daptomycin-induced LCV. Heparin, ceftaroline, vancomycin, and piperacillin-tazobactam were deemed less likely causes of LCV, as their administration was either too brief or distant from the purpura onset at day 21 of daptomycin, which closely matched the 7-21 day window typical for drug-induced LCV. Rare reports of vancomycin- or heparin-induced LCV exist, but the absence of other triggers and daptomycin’s temporal correlation strongly implicate it [18-22]. Applying the Naranjo Adverse Drug Reaction Probability Scale, the temporal association, resolution after daptomycin discontinuation, exclusion of alternative causes, and biopsy confirmation yield a score of 6, indicating a probable causal link between daptomycin and LCV [23]. Based on the American College of Rheumatology (ACR) criteria for diagnosing hypersensitivity vasculitis, which incorporates drug-induced LCV, our patient met the clinical and histopathological diagnostic criteria, as summarized in Table 2 [24].

**Table 2:** American College of Rheumatology (ACR) criteria for hypersensitivity vasculitis. ACR: American College of Rheumatology [24]

Criterion (+3/5)	Definition	Met by This Case? (+5/5)
Palpable purpura	Raised, non-blanching purpura	Yes
Age > 16 years	Patient's age is over 16	Yes
Biopsy showing vasculitis	Neutrophilic infiltration with fibrinoid necrosis	Yes
Maculopapular exanthema	Present on bilateral lower extremities	Yes
Medication exposure	Drug within 7-21 days of onset	Yes

Management of LCV involves a thorough evaluation to identify potential triggers, including newly initiated medications, recent infections, or underlying malignancies. In cases of drug-induced LCV, prompt discontinuation of the offending agent typically results in clinical resolution within days to weeks. However, in severe or life-threatening cases, systemic corticosteroids may be warranted to control inflammation. In rare, refractory cases, immunosuppressive agents, such as methotrexate, azathioprine, mycophenolate mofetil, dapsone, cyclophosphamide, or IV immunoglobulin, may be required [2,25]. It is crucial to accurately identify the underlying etiology of LCV, as the consequence of missing a malignancy trigger or initiating immunosuppressive therapy in a patient with an infectious etiology may be devastating [25].

## Conclusions

This case emphasizes the importance of maintaining a high index of suspicion for LCV as a potential adverse drug reaction, especially in patients presenting with purpuric skin lesions 7-21 days after initiation of a new medication. Given the absence of previously reported cases of daptomycin-induced LCV, this case provides new insights into the spectrum of daptomycin-associated adverse effects. This highlights the importance of early recognition and prompt discontinuation of the inciting agent to achieve favorable clinical outcomes. Given the rarity of daptomycin-induced LCV, further research and pharmacovigilance are warranted to elucidate its underlying pathophysiology and to enhance awareness among clinicians regarding its potential cutaneous adverse effects.
